# Classification of Subcortical Vascular Cognitive Impairment Using Single MRI Sequence and Deep Learning Convolutional Neural Networks

**DOI:** 10.3389/fnins.2019.00627

**Published:** 2019-06-19

**Authors:** Yao Wang, Danyang Tu, Jing Du, Xu Han, Yawen Sun, Qun Xu, Guangtao Zhai, Yan Zhou

**Affiliations:** ^1^Department of Radiology, Renji Hospital, School of Medicine, Shanghai Jiao Tong University, Shanghai, China; ^2^Institute of Image Communication and Network Engineering, Shanghai Jiao Tong University, Shanghai, China; ^3^Department of Neurology, Renji Hospital, School of Medicine, Shanghai Jiao Tong University, Shanghai, China

**Keywords:** subcortical ischemic vascular disease, convolution neural network, cognitive impairment, deep learning, magnetic resonsnce imaging

## Abstract

Deep learning has great potential for imaging classification by extracting low to high-level features. Our aim was to train a convolutional neural network (CNN) with single T2-weighted FLAIR sequence to classify different cognitive performances in patients with subcortical ischemic vascular disease (SIVD). In total, 217 patients with SIVD [including 52 with vascular dementia (VaD), 82 with vascular mild cognitive impairment (VaMCI), and 83 with non-cognitive impairment (NCI)] and 46 matched healthy controls (HCs) underwent MRI scans and neuropsychological assessments. 2D and 3D CNNs were trained to classify VaD, VaMCI, NCI, and HCs based on FLAIR data. For 3D-based model, the loss curves of the training set approached 0.017 after about 20 epochs, while the curves of the testing set maintained at about 0.114. The accuracy of training set and testing set reached 99.7 and 96.9% after about 30 and 35 epochs, respectively. However, the accuracy of the 2D-based model was only around 70%, which performed significantly worse than 3D-based model. This experiment suggests that deep learning is a powerful and convenient method to classify different cognitive performances in SIVD by extracting the shift and scale invariant features of neuroimaging data with single FLAIR sequence. 3D-CNN is superior to 2D-CNN which involves clinical evaluation with MRI multiplanar reformation or volume scanning.

## Introduction

As the population keeps aging, the social and family burden of cognitive impairment has been gradually increasing (Sun et al., 2011). Vascular cognitive impairment (VCI) is a broad term including mild to severe cognitive impairment associated with or caused by cerebrovascular disease (Hachinski and Bowler, 1993). Subcortical vascular cognitive impairment (SVCI) is the most common form of VCI caused by subcortical ischemic vascular disease (SIVD) with various signs in MRI, including lacunar infarction, white matter hyperintensities (WMH) (also termed white matter lesions or leukoaraiosis), prominent perivascular spaces, cerebral microbleeds, and atrophy. These MRI signs have been recognized as the reflection of major pathologies underlying vascular dementia (VaD) and important causes of age-related cognitive decline (Pantoni, 2010; Roh and Lee, 2014). Both the aging of the population and the high incidence of SIVD in aging subjects led to projections of major growth in the numbers of patients with SVCI over the next 30 years (Gorelick et al., 2011). Previous research emphasized that cognitive impairment may be the earliest, most common and sensitive manifestation of cerebrovascular disease, and proposed it as a prognostic indicator (Ingles et al., 2007). However, early detection of subtle cognitive decline is not that easy. The fact is that VaD is detected at a much later stage when treatment cannot stop or reverse but only slow the progression of cognitive decline. So finding an efficient way to perform early diagnosis of VaD is essential for disease prevention and modifying therapies (Smith and Beaudin, 2018). Nowadays, SVCI diagnosis requires very careful clinical assessments such as patients’ history, physical and neurobiological exams, and a detailed neuropsychological evaluation, which are time-consuming, poorly reproducible and even traumatic. More efficient and convenient methods are required to identify the subgroup of SVCI who rapidly decline.

MRI scans allow the non-invasive and efficient collection of multimodal/multiparametric information about morphometry, microstructure, function, perfusion, and even metabolism of the brain. MRI has become a one-stop shop for the macro-scale study of the brain (Diciotti et al., 2015). Meanwhile, computational approaches for excavating and combining the quantitative, intrinsically multivariate and multiparametric information from MR imaging have recently been explored in the field of neuroimaging, although not in an effective enough way. The key challenge of SVCI diagnosis is to determine the clinical relevance patterns for individual patients, as it is difficult to judge whether the load of SIVD is sufficient to result in corresponding clinical cognitive syndromes such as VaMCI or VaD. Recent criteria from the International Society for Vascular Behavior and Cognitive Disorders proposed that one large infarct or hemorrhage, a strategic infarct or hemorrhage, multiple lacunes, or extensive and confluent WMH of vascular origin may be sufficient (Sachdev et al., 2014). However, there is little validation of these thresholds. Besides, most of the existing researches on SVCI diagnosis focused on binary classification problems, i.e., differentiating SVCI subjects from healthy controls (HCs) (Wang et al., 2017). However, for a more precise diagnosis, we need to distinguish among multiple SVCI stages, which makes it a multi-class classification problem.

Previous studies made efforts to address this challenging task by traditional machine learning using multimodal MRI data, including diffusion and morphometry features, and eventually proved the usefulness of machine learning techniques in discriminating HCs from patients suffering from VaD and its prodromal stage: vascular mild cognitive impairment (VaMCI) (Diciotti et al., 2015), which represents a transitional state between non-cognitive impairment (NCI) and dementia. Another study implemented SVM-based machine learning strategy for discrimination between SVCI patients with different cognitive performances through predefined feature vectors extracted from diffusion tensor imaging (DTI) data alone (Ciulli et al., 2016). However, the sensitivity (72.7–89.5%), specificity (71.4–83.3%), and accuracy (77.5–80.0%) are not high enough, probably due to the limited generalization ability of the artificial features. Additionally, extracting those features requires a lot of time, money, and manpower such as human experts. With the rapid development of deep learning algorithms, we can now extract numerous features directly from the images without the engagement of human experts (Ji et al., 2013). Deep learning technologies have been proved to be efficient in various medical image analysis tasks such as MRI, CT, X-ray, mammography, ultrasound, and microscopy (Hamidian et al., 2017; Jyoti and Yanqing, 2018). Deep learning models showed excellent performances in organ and substructure segmentation, disease detection and classification in some areas of pathology, brain, lung, abdomen, cardiac, breast, bone, retina, etc (Ji et al., 2013; Dou et al., 2016; Hamidian et al., 2017; Lao et al., 2017). Classification of such imaging and clinical data were challenging and the most problematic part has always been the selection of the most discriminative features. With the rapid progress of this field, convolutional neural network (CNN) has been utilized in many medical fields, such as in the classification of Alzheimer’s brains and healthy brains and the prediction of underlying molecular genetic mutation status in gliomas (Sarraf and Tofighi, 2016; Chang et al., 2018), which demonstrated that CNNs are capable of learning key imaging components without prior feature selection or human-directed training.

In particular, to our knowledge, no studies have been reported using CNN-based deep learning technique to develop a diagnosis model based on single FLAIR sequence for recognizing SIVD from normal subjects, and discriminating different cognitive performances within the SIVD group. Our hypothesis is that 3D-CNN would show ideal performance. This approach enables us to expand our methodology to predict more complicated systems.

## Materials and Methods

### Participants

In total, 217 subjects with SIVD were recruited from patients admitted to the Neurology Department of Renji Hospital from July 2012 to January 2018. 46 matched HCs were recruited from the community through advertising. SIVD can be defined as subcortical WM hyperintensity on T2-weighted images with at least one lacunar infarct, in accordance with the criteria suggested by Galluzzi et al. (2005). All participants received baseline evaluation, including complete sociodemographic and clinical (cognitive, behavioral, neurological, functional, and physical) data collection. Patient histories were collected from knowledgeable subjects, usually from their spouses. All patients underwent laboratory examinations and conventional MRI for routine investigation of dementia (Wang et al., 2017).

The exclusion criteria were cerebral hemorrhages, cortical and/or corticosubcortical non-lacunar territorial infarcts and watershed infarcts, specific causes of white matter lesions (e.g., multiple sclerosis, sarcoidosis, and brain irradiation), neurodegenerative disease (including AD and Parkinson’s disease), and signs of normal pressure hydrocephalus or alcoholic encephalopathy. Patients with a low education level (<6 years), severe depression [Hamilton Depression Rating Scale (HDRS) ≥ 18], other psychiatric comorbidities or severe cognitive impairment (inability to perform neuropsychological tests), severe claustrophobia, and contraindications to MRI (e.g., pacemaker, metallic foreign bodies) were also excluded. All the participants had lacunar infarcts, small WMH, and slight atrophy (Wang et al., 2017).

Finally, all SIVD patients recruited were subdivided based on cognitive status into subcortical vascular disease with no cognitive impairment group [NCI group (*n* = 83), VaMCI group (*n* = 82), and VaD group (*n* = 52)]. All the participants were right-handed.

The current study was approved by the Research Ethics Committee of Renji Hospital, School of Medicine, Shanghai Jiao Tong University, China. Written informed consent was obtained from each patient.

### Neuropsychological Assessment

Neuropsychological assessments were performed within 2 weeks of the MRI. All subjects did not suffer a new clinical stroke or TIA between the MRI and assessment. A comprehensive battery of neuropsychological tests was designed based on a review of relevant published reports. These tests are as follows: Trail-Making Tests A and B, Stroop color-word test, verbal fluency (category) test, auditory verbal learning test (short and long delayed free recall), Rey–Osterrieth Complex Figure Test (delayed recall), Boston Naming Test (30 words), Rey–Osterrieth Complex Figure Test (copy), Lawton and Brody’s Activities of Daily Living (ADL) Scale Test, Barthel Index (BI), HDRS, and the Neuropsychiatric Inventory (Wang et al., 2017).

To assess the subjects’ cognitive statuses, the scores for each measure of normal-aged patients in Shanghai, China, were used as the normal baseline (norms). Cognitive dysfunction was defined as −1.5 SD in at least one neuropsychological test. According to the AHA Statement on Vascular Contributions to Cognitive Impairment and Dementia (Gorelick et al., 2011), VaD diagnosis was based on a decline in cognitive function from a prior baseline and a deficit in performance in ≥2 cognitive domains that were of sufficient severity to affect the subject’s ability to perform daily activities, which were independent of the motor/sensory sequelae of the vascular event. VaMCI diagnosis was based on the following criteria: (1) ADL could be normal or mildly impaired, (2) does not meet criteria for dementia, and (3) mild quantifiable cognitive impairment within one or more domains (i.e., attention, executive function, memory, language, and visuospatial function). Functional ability was assessed using BI and Lawton and Brody’s ADL scales. However, because most patients with cognitive impairment due to cerebrovascular disease have some degree of disability, the study carefully excluded those with disability due to cognitive damage and motor sequelae using cognitive impairment history and clinical judgement. NCI was defined as subcortical vascular disease with no cognitive impairment, which means the patients’ scores in all neuropsychological tests were within the normal range (<−1.5 SD) (Wang et al., 2017).

### MRI Protocol

MRI was performed with the SignaHDxt 3T MRI scanner (GE Healthcare, United States). An eight-channel standard head coil with foam padding was used to restrict head motion. In addition to conventional brain MRI plain scanning, T2-fluid attenuated inversion recovery sequences (FLAIR) with high resolution were acquired as follows: TE = 150 ms, TR = 9075 ms, TI = 2250 ms, FOV = 256 mm × 256 mm, matrix = 128 × 128, slice thickness = 2 mm, number of slices = 66.

### MRI Pipeline

The pipeline of the proposed method consists of two major parts, data preprocessing and disease classification with CNN-based deep learning strategy. Data preprocessing is designed to extract Region of Interests (ROI) from raw MRI images since the raw ones consist of too many irrelevant data. Then, the processed images are fed to the CNNs we proposed.

### Data Preprocessing

**FIGURE 1:**
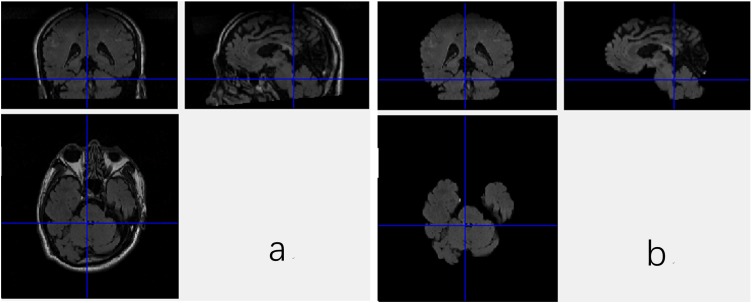
Three views of the MRI data with the non-brain tissues **(a)** and the data without non-brain tissues **(b)**.

DICOM images of FLAIR were firstly transformed into Mat format with a Matlab program, which stores only meaningful volumes for deep CNN and discards the redundant data. Then, the data in Mat format assume the shape of m^∗^n^∗^l^∗^c, where the m, n, l, and c denote length, width and depth as well as number of color channel, respectively. In this work, the m, n, l, and c are equal to 256, 256, 66, and 1, respectively. Brain Extraction Tool (BET) in the FMRIB Software Library (FSL), which is a comprehensive library of analysis tools for FMRI, MRI, and DTI brain imaging data, is employed to extract the brain tissues from the original images, since non-brain tissues may cause unexpected classification results. As a wildly used brain-extraction tool, FSL-BET can easily remove all non-brain tissues from raw images by optimizing the fractional intensity threshold and reducing image bias as well as residual neck voxels. In this work, we set the threshold at a constant, 0.5. The results are shown in Figure 1.

Nevertheless, even when the non-brain tissues are wiped off from the original MRI images, the data are still not able to be fed to the CNN. As shown in Figure 1, the FSL-BET tool can ideally extract the brain-tissues from the original data, but too many zero-values are left in the image. These meaningless zero-values will not only increase the computational expense, but also have a negative impact on the final result. There are basically two strategies to solve this problem, simply downsampling and then rescaling the image to a smaller size or extracting the target region needed. In this work, we used the latter one to get a better result since the first one would reduce the accuracy of classification.

**FIGURE 2:**
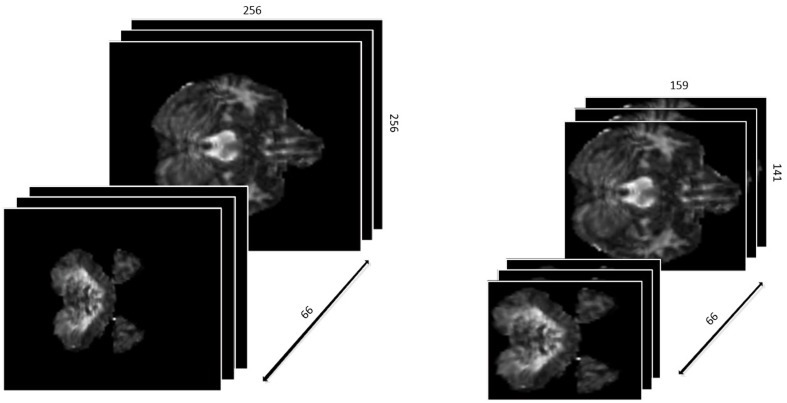
The 3D-shape data before and after preparation.

First, we defined the region with non-zero values in each slice as a ROI. Different slices have different ROIs. To keep the data as a cubic shape, we found the ROI with maximal are MROI in all slices, whose vertex coordinates are (49,60), (207,200), (49,200), (207,200), respectively. Then, each slice was tailored to MROI without changing the positional relationship. With the volume of data reduced from 256^∗^256^∗^66 to 159^∗^141^∗^66, not only it is much easier to train the networks, but the accuracy of classification is greatly improved since the overfitting problem is resolved. The results are shown in Figure 2.

### Statistical Analysis

#### Convolutional Neural Networks

The traditional machine leaning method requires manually extracting the features using prior knowledge, and then trying to find the linear or non-linear relationship between these features and target label, which is quite challenging for researchers. Firstly, it requires a very large amount of knowledge to designate the features to be selected. In addition, large numbers of features are time consuming. This problem has been solved thanks to the development of deep learning. Recent studies have shown the great capacity of deep learning in computer vision tasks, such as image classification and object detection. With a deep structure, it is easy to get a larger number of features, which are also are commonly more complex and advanced.

**FIGURE 3:**
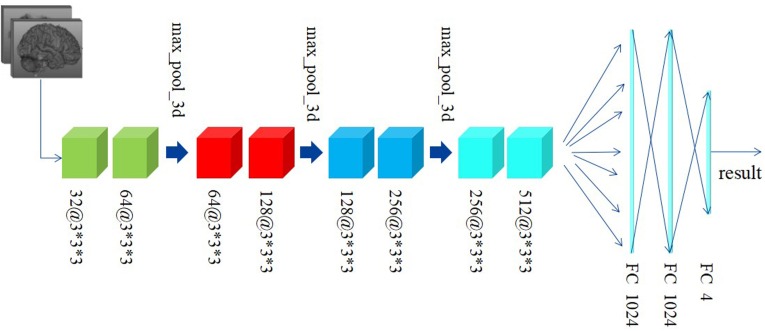
The construction of the 3D-based convolutional network we proposed.

Differently from the traditional image classification task, the data after preprocessing has a 3D construction, which consists of 66 slices and each slice has a shape of 159^∗^141, as described above. As a result, the traditional 2D-based convolutional networks, such as AlexNet, VGG, and ResNet, etc., may be not suitable for our task, as such models have no capacity to extract the structural features among the correlative slices. Considering this situation, we proposed a 3D-based convolutional network, as shown in Figure 3.

Figure 3 shows the custom 3D-based architecture in this study. A 3D-convolutional layer is represented as the cube, and we used the text [channel numbers @ filter size] to indicate the channel numbers and filter size in each convolutional layer. For example, 32@3^∗^3^∗^3 means: (1) the output of this convolutional layer contains 32 channels; (2) the filter size is 3^∗^3^∗^3, which means the filter has a cubic shape, receiving the data from three adjacent slices. This differs from a traditional 2D-based filter that only receives data from only one slice at a time.

Preprocessed data with resolution of 159^∗^141 and totally 66 slices are fed to our custom network. Generally, since the features extracted at a deeper layer are more complex and abstract, the number of channels would correspondingly and gradually increase. According to this principle, the channel’s number in convolutional layers in our custom network is gradually increased from 32 to 512. Subsequently, there are eight convolutional layers with different numbers of input and output channels followed by two fully connected (FC) layers activated by linear function and one FC layer activated by softmax. The Rectified Linear Unit (RELU) function, defined as the Eq. 1, is used as the activation function. In addition, the data every two convolutional layers is downsampled by a max-pooling layer with a filter in the size of 2^∗^2^∗^2 and stride of 2^∗^2^∗^2. The cross-entropy loss function, defined as Eq. 2, is used to minimize the loss function along with the Stochastic Gradient Descent (SGD) optimizer.

(1) R(x)=max(x, 0)

(2) $$\text{loss}=-\sum_{i=1}^{N}y_{i}\log y_{i}^{\wedge }+(1-y_{i})\log(1-y_{i}^{\wedge })$$

To address the overfitting issues, the batch normalization method is performed before each activation function. Learning rate is lowered as the training process progresses, having been initialized as 1e-2. The decayed learning rate is defined as Eq. 3, where the decay step is equaled to 300.

(3) $${\text{lr}}_{\text{new}}={\text{lr}}_{\text{old}}*{\text{lr\_decay}}^{\frac{\text{global\_step}}{\text{decay\_step}}}$$

#### Experimental Settings

We implemented our model with keras (with TensorFlow as backend). All the experiments were performed on a workstation with Inter Core i7 processor and three NVIDIA 1080 Ti GPUs.

We used 75 and 25% of the data for training and testing and the accuracy was employed to evaluate our custom model. In addition, we employed k-fold cross validation for the training set. We divided the training dataset into 10 equal shares. For each training time, nine shares were used for training and the other one was used for validation. Finally, the mean of the results of 10 training sessions represented the accuracy of the training set.

In addition to accuracy, we also introduced three other significant metrics vastly applied to evaluate a classification model, including recall (R), precision (P), and F1 score. Using TP, FN, FP, NP to denote the number of the positive samples classified to positive, the positive sample classified as negative, the negative samples classified as positive and the negative samples classified as negative, respectively. Then, the R, P, and F1 score can be defined as:

(4) $$\text{Recall}=\frac{\text{TP}}{\text{TP}+\text{FP}}$$

(5) $$\text{Precision}=\frac{\text{TP}}{\text{TP}+\text{FN}}$$

(6) $$\frac{2}{\text{F}1}=\frac{1}{\text{R}}+\frac{1}{\text{P}}$$

Differently from the binary classification task, our custom model has to figure out four different categories. Therefore, when we calculate these metrics for each category, the positive samples denote the target category and the other three categories would be the negative samples. For example, when it comes to MCI, we remark the MCI as positive samples and the NCI, dementia, and contrast are all remarked as negative samples.

## Results

### 2D or 3D CNN

For traditional image classification task, 2D convolutional kernels are more widely applied, since we normally regard an image as 2D matrix without considering the discrete color channel. However, quite differently from a traditional digital image, even without considering the color channel, the MRI image still consists of three dimensions. More than the width and length of a traditional image, we have to take the depth into account. If we just simply apply traditional 2D convolutional kernels, the model will have no ability to extract the spatial features among adjacent slices. To compare the different performances of different models with 2D and 3D convolutional kernels, we also apply the VGG-16, a wildly used and excellent 2D-based CNN model for natural image classification task, to classify the SVCI disease. The results obtained are shown in Table 1.

**Table 1:** Average evaluation metrics of 3D-based convolutional networks compared to the traditional 2D-based convolutional networks.

Labels	Classifier	Recall	Precision	F1-score
Contrast	2D	0.61	0.62	0.61
	3D	0.96	0.94	0.95
NCI	2D	0.59	0.54	0.56
	3D	0.93	0.9	0.91
MCI	2D	0.57	0.55	0.56
	3D	0.94	0.93	0.93
Dementia	2D	0.62	0.64	0.63
	3D	0.95	0.91	0.93

### Loss and Accuracy

**FIGURE 4:**
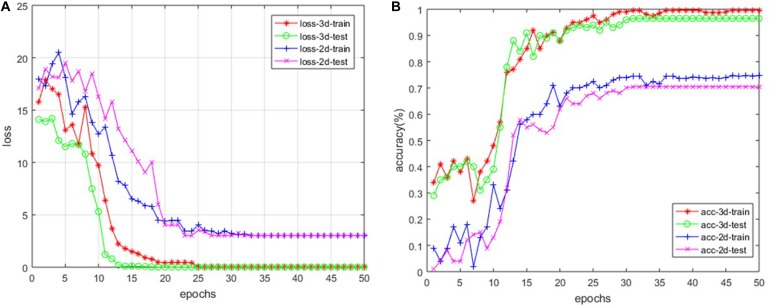
The loss curve **(A)** and the accuracy curve **(B)** of both 2D- and 3D-based models.

For our custom 3D-based deep CNNs, we trained the model for a total of 50 epochs with random initialization. Meanwhile, we fine-tuned the 2D-based VGG-16 model, which has been pre-trained with ImageNet. After every epoch, we recorded both the values of accuracy and loss function on the training dataset as well as the testing dataset. Figure 4 shows the loss and accuracy curves of our model for SVCI classification.

Our findings are that the 3D-based model performs much better than 2D-based model. The main reason is that the 2D-based model doesn’t have the ability to retract the structure features among the adjacent slices, as described above. For the 3D-based model, the loss curves of the training set approach 0.017 after about 20 epochs, while the curves of the testing set are stable at about 0.114. The loss curve continues to decline and stabilize, demonstrating that the model works and there is no slightly overfitting. The accuracy of training set and testing set reached 99.7 and 96.9% after about 30 and 35 epochs, respectively. However, the accuracy of 2D-based model is only around 70%, which is much more poorly than the 3D-based model’s performance.

## Discussion

In this paper we presented deep learning models for classification of different cognitive performances in SIVD, which overcome the limited feature set extracted by conventional handcrafted methods. Using 3D-CNN, we were able to accurately recognize SIVD from normal subjects, and discriminate within the SVCI group between patients with different cognitive performances, based on single FLAIR sequence. Higher-order deep features were extracted and included in our deep learning model. The accuracy of the training set and the testing set reached 99.7 and 96.9% after about 30 and 35 epochs, respectively. This result is not surprising, as deep features reflect higher-order imaging patterns and capture more imaging heterogeneity compared with low-level shape, intensity, and texture features.

Nowadays, neuroimaging is an indispensable part of clinical assessments and has an increasingly important role for early detection of SVCI. Researchers have been devoting their efforts to neuroimaging techniques and computational approaches for the qualitative and quantitative analysis of pathological brain changes related to SIVD. Prior classic machine-learning approaches for linking imaging features to cognitive decline in SIVD patients have relied on human-derived feature extraction. For example, Ciulli et al. (2016) employed a functional tree classifier for the classification of healthy subjects and VaMCI and the prediction of cognitive performance by combining DTI parameters with brain morphometry. Compared with previous methods that employed low-level hand-crafted descriptors, our method can take full advantage of spatial contextual information in MR volumes to extract more representative high-level features with FLAIR data alone, and hence achieve a much better detection accuracy.

In the medical image processing field, especially for 3D data computing tasks, 3D CNNs hold promising potentials but have not been well explored in SIVD subjects yet. However, 3D CNNs based on MR images have been widely used in the field of other medical imaging, including Alzheimer’s disease (Sarraf and Tofighi, 2016; Jyoti and Yanqing, 2018), detection of cerebral microbleeds (Dou et al., 2016), prediction brain age (Cole et al., 2016), classifying genetic mutations in glioma (Lao et al., 2017). In particular, the application of 3D CNN in Alzheimer’s disease has reference value for the subdivision diagnosis of SIVD. For neuroimaging data, deep learning algorithms have potential of discovering latent or hidden representations and efficiently capture the disease-related pathologies. Payan and Montana (2015) trained sparse autoencoders and 3D CNN models for AD diagnosis. They also developed a 2D CNN model and found that the 3D approach has a superior performance for the 3-way comparison, as well as the AD vs. MCI and HC vs. MCI comparisons. In this paper, we also used a 2D-based CNN model to classify the SVCI disease. Finally, results proved that 3D-based CNN model performed much better than 2D-based model for SVCI disease classification. This result is similar to a previous AD study. The main reason is that MRI imaging data consists of numerous slices which have a continuous spatial positional relationship, which is hard for a 2D-based network like VGG-16 to extract. This prompted the proposal for clinical evaluation with MRI multiplanar reformation or volume scanning to improve classification accuracy. Last but not least, we chose FLAIR sequence as raw data for the deep learning of SIVD because of the high-contrast display and maximum reflection of pathological changes, which is different from studies of AD usually using T1-weighted images as raw data.

Although the proposed method has achieved an appealing performance with a high accuracy, there are still several limitations. First, this is a retrospective study with a relatively small sample size. In the future, large-scale multicenter studies are required to fully assess the generalization ability of the model. Second, the interpretation of the association between the deep features and the cognitive performances remain challenging. It might be related to complex biological processes. Further studies are needed to establish a rationale to explain the correlation between deep imaging features and cognitive performances.

## Conclusion

We presented an efficient and robust method to automatically recognize SIVD from normal subjects and discriminate within the SIVD group between patients with different cognitive performances by 3D CNN-based deep learning. This provides a global perspective method for clinical evaluation of SIVD from convenient single MRI scan.

## Data Availability

The datasets generated for this study are available on request to the corresponding author.

## Ethics Statement

This study was carried out in accordance with the recommendations of Research Ethics Committee of Renji Hospital, School of Medicine, Shanghai Jiao Tong University, China, with written informed consent from all subjects. All subjects gave written informed consent in accordance with the Declaration of Helsinki. The protocol was approved by the Research Ethics Committee of Renji Hospital, School of Medicine, Shanghai Jiao Tong University, China.

## Author Contributions

YZ, GZ, and QX conceived and designed the experiments. YW, DT, JD, XH, and YS performed the experiments. YW, DT, and JD analyzed the data and wrote the manuscript. XH and YS contributed materials, analysis tools, and figures. All authors listed have made a substantial, direct and intellectual contribution to the work, and approved it for publication.

## Conflict of Interest Statement

The authors declare that the research was conducted in the absence of any commercial or financial relationships that could be construed as a potential conflict of interest.
